# Direct evidence of metal–ligand redox processes in positive electrodes during lithium-based battery operation

**DOI:** 10.1038/s41565-026-02189-y

**Published:** 2026-06-09

**Authors:** Galo J. Páez Fajardo, Daniela E. Dogaru, Hrishit Banerjee, Muhammad Ans, Matthew J. W. Ogley, Veronika Majherova, Gerard Bree, Innes McClelland, Shohei Hayashida, Pascal Puphal, Masahiko Isobe, Bernhard Keimer, Pardeep K. Thakur, Tien-Lin Lee, Dave C. Grinter, Pilar Ferrer, Serena A. Cussen, Matthias Hepting, Louis F. J. Piper

**Affiliations:** 1 WMG, University of Warwick, Coventry, UK; 2 The Faraday Institution, Quad One, Harwell Science and Innovation Campus, Didcot, UK; 3 School of Science and Engineering, University of Dundee, Dundee, UK; 4 School of Metallurgy and Materials, University of Birmingham, Edgbaston, Birmingham, UK; 5 School of Chemistry, University College Dublin, Belfield, Dublin, Ireland; 6 Max Planck Institute for Solid State Research, Stuttgart, Germany; 7 Diamond Light Source Ltd, Harwell Science and Innovation Campus, Didcot, UK

**Keywords:** Batteries, Electronic properties and materials, Materials for energy and catalysis, Theoretical chemistry

## Abstract

Describing lithium-based battery positive electrodes based on different transition metal or oxygen-redox regimes can cause confusion in understanding metal–ligand hybridization, oxygen dimerization and degradation processes. Therefore, it is urgent to investigate the electronic structure of these materials and identify the role each cation and anion has in charge compensation at the subnanoscale. Here, using X-ray resonance photoemission spectroscopy, single-impurity Anderson models, spectral simulations and theoretical calculations, we examine redox mechanisms in positive electrodes during lithium-based battery operation. This approach reconciles the redox description of two positive electrode active materials—LiMn_0.6_Fe_0.4_PO_4_ and LiNiO_2_—in terms of varying degrees of charge transfer using the Zaanen–Sawatzky–Allen framework. In LiMn_0.6_Fe_0.4_PO_4_, the lack of strong hybridization indicates that the capacity results from the depopulation of metal 3*d* states, that is, conventional metal redox. However, in cells with LiNiO_2_-based positive electrodes, negative charge transfer dominates, and redox occurs through the formation and elimination of ligand-hole states. These results clarify the role of oxygen in Ni-rich systems and provide a framework to explain how the charge/discharge capacities are linked to oxygen-dominated states in highly covalent systems, without the need to consider oxygen dimerization.

## Abstract (web summary)

Redox electrons in lithium-based battery positive electrodes originate from hybridized orbitals. Combined characterization methods show that across the 3*d* transition metal series, their origin shifts, with conventional electron counting valid in early transition metal compounds and ligand-hole redox dominating in late transition metal oxides.

## Main

Oxygen loss limits the battery performance of both Li^+^-excess and stoichiometric Ni-rich positive electrodes^1–3^. Efforts to suppress it are often conflated with oxygen redox, commonly associated with oxygen dimerization and O_2_ formation in Li-excess compounds^4^. This has driven a broader re-examination of oxygen participation in redox, leading to two distinct formalisms: non-bonding O2*p* orbitals and metal–ligand hybridization^5^. The former is widely invoked to explain O_2_ formation observed in resonant inelastic X-ray scattering^4^, yet compounds that challenge this interpretation still exhibit oxygen-dimer features generated at high cut-off potentials^6–9^. Moreover, recent studies question whether these resonant inelastic X-ray scattering signatures correlate with reversible capacity, suggesting instead that they may arise from X-ray-induced core excitation that artificially generates molecular O_2_ signals^10^. Alternative structural pathways for O_2_ formation have also been proposed^11,12^. Within this conflicting landscape, oxygen participation via hybridization is increasingly supported, with beyond-density-functional-theory (DFT) studies, indicating its contribution from the onset of delithiation^13^, corroborated by operando X-ray measurements^14^. These discrepancies highlight the need to directly resolve oxygen’s electronic role during redox.

1 $${\rm{L}}{{\rm{i}}}^{+}{\rm{N}}{{\rm{i}}}^{3+}{{\rm{O}}}_{2}^{2-}\to {\rm{L}}{{\rm{i}}}^{+}+{{\rm{e}}}^{-}+{\rm{N}}{{\rm{i}}}^{4+}{{\rm{O}}}_{2}^{2-},$$

2 $${\mathrm{Li}}^{+}{\mathrm{Fe}}^{2+}0.4{\mathrm{Mn}}^{2+}0.6{\left({\mathrm{PO}}_{4}\right)}^{3-}\to {\mathrm{Li}}^{+}+{{\rm{e}}}^{-}+{\mathrm{Fe}}^{3+}0.4{\mathrm{Mn}}^{3+}0.6{\left({\mathrm{PO}}_{4}\right)}^{3-}.$$

Equation (1) describes the end-points of LiNiO_2_ (LNO) delithiation, where charge compensation nominally raises the Ni formal oxidation state. The interpretation of this depends on the underlying electronic structure, and competing frameworks arise accordingly. In the classical picture, oxidation is tied to changes in transition metal (TM) 3*d* charge density under an assumption of fully ionic TM–O bonds, leading to discrete changes in 3*d* occupancy^15–17^. LiMn_0.6_Fe_0.4_PO_4_ (LMFP64) exemplifies this behaviour, where equation (2) yields a Fe^2+^|Fe^3+^ redox pair that directly reflects variations in Fe3*d* occupancy. By contrast, although the Ni formal oxidation state increases in LNO, it does not correspond to discrete changes in Ni3*d* occupancy. This reflects the breakdown of the ionic picture, as bond disproportionation^18^, intersite charge fluctuations^19^ and strong metal–ligand hybridization^20^ give rise to a more complex electronic structure below the nanoscale.

This complexity exposes a key limitation of current approaches. Most studies infer redox mechanisms indirectly by probing core-level or unoccupied states rather than the orbitals directly involved in charge compensation. Although sufficient for ionic systems, this strategy can be misleading in compounds such as LNO. For example, during electrochemical Li-ion deintercalation, hard X-ray photoelectron spectroscopy (XPS) shows narrowing but no energy shift of Ni2*p* peaks^21^, whereas Ni K-edge X-ray absorption near-edge spectroscopy (XANES) reveals that the absorption edge shift halts despite continued capacity extraction^8,22,23^. Raman spectroscopy indicates evolving Ni–O covalency^19^, and Ni L-edge resonant inelastic X-ray scattering suggests a dominant Ni3*d*^8^ character rather than the expected 3*d*^7^ configuration^19^. Although theoretical studies have addressed these complexities^24,25^ and recent frameworks achieve sensitivity to such electronic structure from first principles^26^, direct experimental validation remains limited. Together, these inconsistencies underscore the need for methods that directly resolve the character and evolution of redox-active orbitals.

Here we implement a methodology that directly resolves the character of the orbitals involved in the charge compensation, which reside at the top of the valence band. The approach combines electronic structure calculations, TM L-edge X-ray absorption spectroscopy (XAS), and resonant photoemission spectroscopy (RPES)^27,28^ to determine how configurations such as orbital symmetry (t_2g_/e_g_), elemental contributions, metal–ligand hybridization and more complex phenomena including intersite charge fluctuations and bond disproportionation govern redox mechanisms. Single-impurity Anderson (SIA) calculations of TM L-edge XAS spectra^29^ identify these underlying configurations and define the corresponding excitation-energy range. Performing RPES across this SIA-informed excitation energy range selectively enhances respective metal 3*d* and oxygen 2*p* spectral contributions. By correlating these orbital fingerprints with positive electrodes at different states of charge (SoC), this combined experiment–theory framework reveals how fundamental electronic structure evolves during charge compensation pathways and how it governs redox mechanisms across materials.

We apply this approach to two canonical systems: LMFP64 (representative of ionic redox) and LNO (which exhibits more complex electronic behaviour). Both are studied in non-aqueous Li metal coin cells under relevant electrochemical conditions^23,30^. We find that LMFP64 follows the classical redox mechanism, consistent with an electronic structure well described by the ionic picture, driven by strong inductive effects from the polyanion framework^31^. By contrast, LNO exhibits a fundamentally different redox mechanism governed by bond disproportionation, intersite charge fluctuations and metal–ligand hybridization, consistent with a ligand-hole-driven description^13,14^. These results provide a direct insight into the electronic structure and a pathway to understanding oxygen redox.

## Results

### Classical redox model in LMFP64

**Fig. 1:**
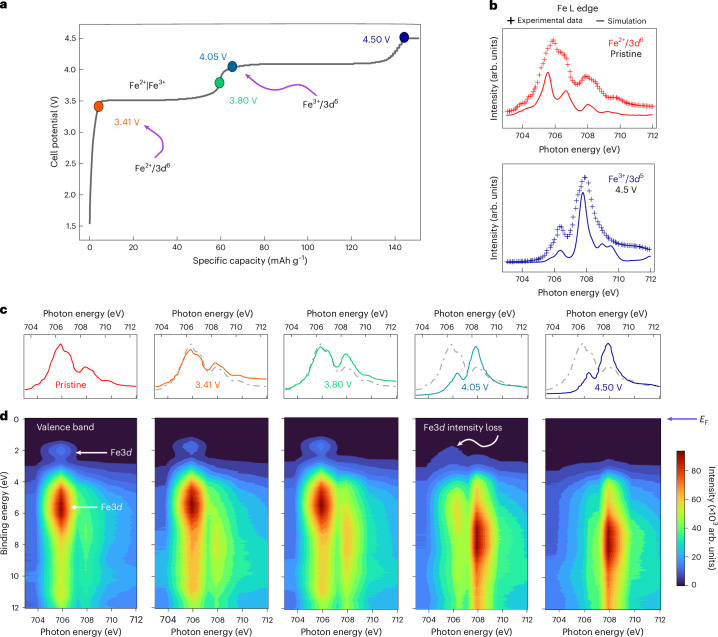
Resonant photoemission probe of LMFP64 redox during charging. **a**, Electrochemical data of Li||LMFP64 coin cells during *C*/20 (7.93 mA g^−1^) charge (at 25 °C) with circular shapes indicating the cell potential points at which we disassembled the coin cells for the XAS and RPES measurements. **b**, Comparison of simulated spectra of Fe L-edge XAS with experimental data. **c**, Fe L-edge XAS measurements in the TEY mode of the LMFP64 electrodes at 3.41, 3.80, 4.05 and 4.50 V along with pristine electrodes to identify X-ray photon energies for the RPES studies. These cell potentials corresponding to 3.45%, 41.38%, 44.83% and 100% SoC (100% SoC of ~145 mAg h^−1^). **d**, Heat maps of the RPES experiments showing the evolution of the electronic structure as a function of delithiation in LMFP64. Binding energy is shown on the *y* axis of the RPES maps. The Fermi level *E*_F_ is at the zero binding energy mark (blue arrow). **c** and **d** share the same *x* axis.

The Fe redox mechanism in LMFP64 represents the archetypal conventional model in which electrochemical delithiation corresponds to initial electron depopulation from Fe3*d* orbitals. We use this material as a reference to establish spectral fingerprints of the classical redox picture. Non-aqueous Li||LMFP64 coin cells were charged to the targeted potentials (Fig. 1a). A slow *C*/20 rate (7.93 mA g^−1^) was used to minimize fast-rate-induced structural transformations (2C ≈ 317.2 mA g^−1^)^32^ and intercalation artefacts^33^, ensuring negligible polarization and a close approximation to open-circuit conditions^3^.

Figure 1b (top) compares the experimental Fe L-edge XAS in the total electron yield (TEY) mode of the pristine electrode (no cycling or electrolyte exposure) with SIA simulations. Calculations performed without Fe3*d*–O2*p* hybridization (ionic limit) and with a 3*d*^6^ configuration (Fe^2+^) agree well with the experimental spectrum. Simulating the charged state (4.5 V) by reducing the occupancy to 3*d*^5^ and maintaining this description similarly reproduces the measured spectrum. Together, these results indicate that LMFP64 redox proceeds via a reduction in Fe3*d* electron count from 3*d*^6^ to 3*d*^5^ during Fe^2+^|Fe^3+^ oxidation.

This electron count reduction can be directly probed using RPES performed across the Fe L-edge 704–709 eV range, where the resonant excitations is strongly dominated by Fe3*d* character. Under these conditions, RPES selectively enhances Fe3*d* contributions within the redox-sensitive orbitals, enabling the direct tracking of how these electronic configurations govern LMFP64’s charge compensation mechanism. RPES measurements (Fig. 1d), taken across the Fe L-edge (Fig. 1c), show a systematic decrease in the Fe3*d* intensity near the ~2-eV binding energy with increasing potential, directly evidencing electron removal during Fe oxidation. The dominant spectral changes occur across the Fe^2+^|Fe^3+^ plateau (Fig. 1a) rather than scaling linearly with the extracted capacity, reflecting the multiplet nature of L-edge XAS—and hence RPES—final states (Supplementary Note 1). Nevertheless, these trends remain consistent with operando Fe K-edge XAS measurements on pouch cells under realistic conditions, supporting their relevance to practical redox behaviour (Supplementary Figs. 1–3). These results confirm that in LMFP64, the formal oxidation states map onto discrete changes in Fe3*d* occupancy, validating the classical ionic redox picture.

### LNO: classical redox?

**Fig. 2:**
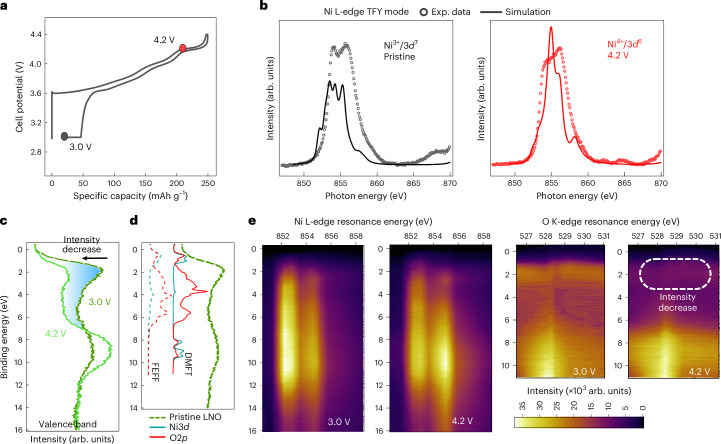
Oxygen orbitals and their involvement in LNO redox. **a**, Electrochemical data of Li||LNO coin cells during *C*/20 (11 mA g^−1^) charge and discharge (at 25 °C), with circular shapes indicating the cell potential points at which we stopped the measurements and disconnected the cells to carry out cells’ disassembly and electrode harvesting for XAS and RPES studies. **b**, Comparison of the simulated spectra of Ni L-edge XAS with experimental data in the TFY mode. **c**, XPS measurements at 1,000 eV of the valence band region for positive electrodes charged at 4.2 V and after discharge to 3.0 V, showing intensity changes that correlate with the corresponding Ni oxidation states at each potential. **d**, DMFT and GW0-based calculations of the valence band density of states, identifying the spectral regions dominated by Ni3*d* and O2*p* character. **e**, RPES at the Ni L-edge, indicating minimal spectral change, consistent with negligible Ni3*d* participation during redox, and RPES at the O K edge, showing a substantial loss of spectral intensity on charge, attributed to the oxidation and depletion of O2*p*-derived ligand-hole states.

If Ni3*d* electron counting governed redox in LNO, Ni L-edge RPES would mirror the behaviour observed in LMFP64. We, therefore, examined this expectation by assembling Li||LNO coin cells and cycled them at *C*/20 (11 mA g^−1^) between 3.0 and 4.2 V (Fig. 2a).

In contrast to LMFP64, Ni L-edge simulations within the ionic picture (that is, without explicit Ni3*d*–O2*p* hybridization) show poor agreement with experiment. Both pristine (3*d*^7^) and charged (3*d*^6^) configurations fail to reproduce the bulk-sensitive Ni L-edge spectra measured in the total fluorescence yield (TFY) mode (Fig. 2b), indicating that LNO redox cannot be described by simple Ni3*d* electron counting and instead reflects a more complex electronic structure.

To resolve this discrepancy, we combine valence-band XPS with theory. Valence-band XPS (Fig. 2c) shows clear evolution of states near ~2 eV between 4.2 V and 3.0 V, identifying these as redox active. Dynamical mean-field theory (DMFT) simulations and GW0 (FEFF software) calculations resolve their character (Fig. 2d). Despite their distinct theoretical frameworks, both consistently assign the valence-band edge to hybridized Ni3*d*–O2*p* states, whereas the Ni3*d* states remain deeper in energy, around 10 eV.

RPES measurements directly validate this picture. Ni L-edge RPES identifies the ~10-eV feature as a cycling-invariant Ni3*d* state, indicating that Ni3*d* occupancy remains largely unchanged across Li||LNO cell potentials in Fig. 2a. The same feature appears in O K-edge RPES, confirming a strong Ni3*d*–O2*p* hybridization. Resonant excitation at the O K-edge pre-edge—enhancing this hybridized character—reveals pronounced changes in the ~2-eV states identified by XPS. Building on the Fe case, where such changes track electron occupancy, the combined Ni L-edge and O K-edge RPES results show that redox in LNO predominantly involves ligand states within hybridized Ni–O orbitals.

Collectively, our results reveal a shift in the redox mechanism—from metal-centred in LMFP64 to ligand-dominated through cooperative Ni–O hybridization. Questions then arise as to why this shift happens and how it influences redox chemistry.

### Electronic structures: from metal centred to ligand hole

Understanding this transition requires moving beyond material-specific observations to a more general description of how the electronic structure evolves with the formal oxidation state. The shift in redox mechanism described in the previous section originates from the evolution of electronic structure with increasing formal oxidation state. As metals are oxidized, increasing electron affinity combined with decreasing ligand electronegativity drives a progressive departure from simple ionic bonding descriptions^16^, leading to increasingly complex electronic configurations. Using our approach, we probe how this evolution manifests spectroscopically, developing a framework to comprehensively assess the LNO redox activity.

To establish this trend, we consider a series of Ni-oxide compounds (LaNiO_2_, NiO, LNO and Li_2_NiO_3_), spanning formal Ni oxidation states from Ni^1+^ to Ni^4+^, respectively. The Ni–O ligand hybridization is minimal in LaNiO_2_ (ref. ^34^) and has a substantial role in NiO (ref. ^35^). LNO is better described with intersite charge fluctuations, explicit TM3*d*–O2*p* ligand hybridization and bond-disproportionate NiO_6_ sites^21^. Li_2_NiO_3_ is the end-point of Li excess and its electronic structure is better described by intersite charge fluctuations and explicit TM3*d*–O2*p* ligand hybridization^36^. This series, therefore, enables us to track how increasing formal oxidation drives a transition from weakly to strongly hybridized electronic structures.

**Fig. 3:**
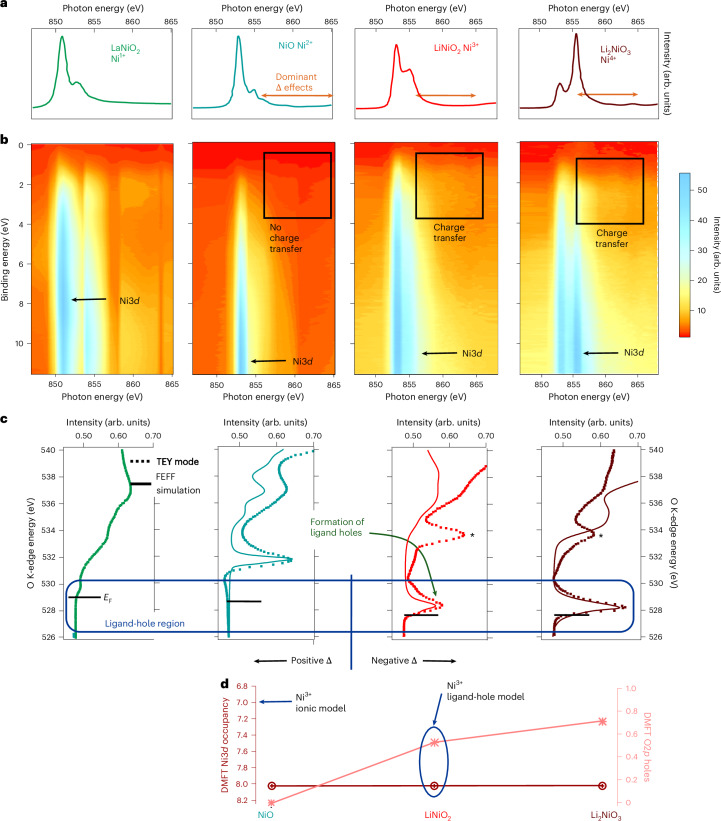
Ni oxides and ligand-hole evolution. **a**, Ni L-edge XAS measurements in the TEY mode of various Ni oxide compounds to identify X-ray photon energies for the RPES studies. **b**, Heat map showing the intensities of the RPES experiments indicating the energies of occupied orbitals and the effects of charge transfer into the Ni3*d* from the ligand orbital. **c**, Unoccupied O2*p* orbitals in the TEY-mode O K-edge experiments (dotted lines) and Green’s-function-based O K-edge simulations (solid lines) with a peak whose intensity increases due to ligand-hole formation as a result of spontaneous charge transfer from the O2*p* ligand to Ni3*d*e_g_ in the negative-Δ regime. Asterisk denotes the typical carbonate (Li_2_CO_3_) surface contamination only affecting high-valence Ni oxides. **d**, DMFT-derived occupancies of the Ni3*d* and O2*p* orbitals correlating with the increasing pre-edge peak in O K edge as a combined signature of ligand-hole creation, dominating the oxidation state evolution of Ni oxides.

Figure 3a shows the TEY-mode Ni L-edge XAS spectra across this series, with the corresponding RPES data shown in Fig. 3b. Because RPES is surface sensitive, the measured Ni L-edge response can conflate electronic features from surface and subsurface regions in Ni oxides that undergo surface reconstruction. We, therefore, first differentiate these contributions. Supplementary Fig. 4 and Supplementary Note 2 identify Ni L-edge surface-related features near ~853 eV, whereas excitations above 854 eV originate predominantly from subsurface regions. We, thus, restrict Ni L-edge RPES excitation to 854–865 eV, defining an energy window that isolates the intrinsic electronic structure and enables the tracking of the formal oxidation-driven evolution across the series.

Within this 854–865-eV window, Ni L-edge RPES reveals the emergence of spectral weight on going from Ni^2+^ (NiO) to Ni^3+^ (LNO) compounds (Fig. 3b, black squares), indicating that this spectral signature tracks the evolution towards more strongly hybridized electronic structures with increasing formal oxidation. To interpret this, we analyse the NiO reference using SIA-based simulations (Supplementary Fig. 5 and Supplementary Note 3). Reproducing the spectral features in this range requires the explicit inclusion of Ni3*d*–O2*p* hybridization (Supplementary Fig. 5b), which enables charge fluctuation from O2*p* to Ni3*d* states through shared hybridized orbitals. In NiO, however, this process is not spontaneous but induced by X-ray excitation—commonly referred to as the Zhang–Rice bound state^35^. Its energetics are described by the charge-transfer energy Δ, which is positive in NiO, indicating that ligand-to-metal charge transfer is not intrinsically favoured. Consequently, NiO lacks intrinsic charge-transfer features in RPES within this window. We, therefore, define the 854–865-eV range as a Δ-dominant region, where RPES selectively probes hybridized states with a spontaneous charge-transfer character, expected to emerge in negative-Δ systems.

The pronounced Ni L-edge RPES features resonating in this Δ-dominant range for LNO and Li_2_NiO_3_ indicate that the transition to the negative-Δ regime occurs between Ni^2+^ and Ni^3+^. This interpretation can be directly tested across a chemically controlled oxidation series. Thin films of the Li_*x*_Ni_1−*x*_O family (*x* = 0 corresponds to Ni^2+^ and *x* = 0.5, to Ni^3+^), designed to emulate this oxidation progression, further support this assignment (Supplementary Note 4). DMFT orbital occupancy analysis on the models of this series (Supplementary Fig. 6) shows that beyond Ni^2+^, spontaneous charge migration onsets at *x* = 0.09 and stabilizes thereafter through the formation of electronic ‘holes’ ($\underline{L}$) in the O2*p* ligand states within hybridized orbitals. Spectral measurements (Supplementary Fig. 6) across the thin-film series validate this picture, revealing the same onset at *x* = 0.09 as a transfer of intensity from occupied hybridized Ni3*d*–O2*p* states to the lowest unoccupied states at the O K-edge pre-peak. The growth of this pre-peak, therefore, provides a direct spectroscopic signature of ligand-hole formation, consistent with previous reports^37,38^.

Applying this framework to Fig. 3b–d, LNO and Li_2_NiO_3_ exhibit clear signatures of negative-Δ systems. Their formal oxidation states exceed Ni^2+^, placing them beyond the positive-to-negative Δ transition threshold and stabilizing ligand holes, as confirmed by DMFT. Experimentally, this manifests as pronounced Ni L-edge intensity in the Δ-dominant region and a systematic growth of the O K-edge pre-edge, consistent with the increase in ligand-hole population predicted by DMFT and reproduced in simulations using the FEFF software. Together, these observations establish the O K-edge pre-edge as a robust descriptor of ligand holes and identify ligand-hole formation as the mechanism governing electronic structure evolution at high Ni oxidation states. The consistency across DMFT, multiplet effects in SIA and real-space correlation in GW0 (FEFF) reconciles these approaches and underscores both the robustness of this description and the central role of ligand holes in determining the behaviour of high oxidation-state Ni systems.

In contrast to the LMFP64 ionic limit, this integrated experimental and theoretical analysis establishes LNO and Li_2_NiO_3_ as prototypical negative-Δ systems, in which ligand holes are present in the pristine state and their formation and annihilation govern evolution across the full Ni^2+^ ↔ Ni^3+^ ↔ Ni^4+^ range.

### Understanding redox in a ligand-hole electrode

The ligand-hole formation is not only intrinsic to the electronic structure but is actively modulated during electrochemical cycling, as exemplified by LNO-based positive electrodes. In Supplementary Fig. 7, as the upper cell potential cut-off increases from 4.2 V to 4.6 V, the O K-edge pre-edge feature exhibits the same intensity growth (Supplementary Fig. 7b), previously associated with ligand-hole formation. This trend mirrors the O K-edge spectral evolution of the Ni oxide series (Fig. 3c) and suggests that the electrochemical charging process across the full potential range of LNO formulated in a composite electrode must be governed by the progressive formation of ligand holes. Resonance in the Δ-dominated range in Ni L-edge RPES measurements (Supplementary Fig. 7c) reveals a spectral reduction of the ligand-hole hybridized orbitals as the cell potential increases. This reduction (Supplementary Fig. 7d, white squares) indicates a progressive electron withdrawal from the ligand-hole states, confirming their direct participation in LNO charge compensation.

To interpret these observations, we first examine bulk-sensitive (TFY-mode) Ni L-edge measurements of pristine LNO electrodes (no cell cycling and no exposure to electrolyte solution), together with Ni L-edge simulations in the relevant Δ regime.

**Fig. 4:**
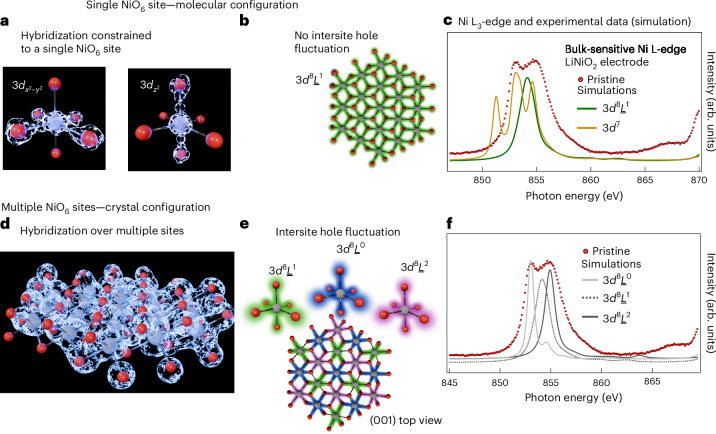
Coexistent Ni sites in LNO. **a**,**b**,**d**,**e**, Space occupied by the hybridized Ni3*d*–O2*p* ligand orbital relative to a single NiO_6_ site (**a**) or over multiple sites (**d**) has an effect on the type of hole configuration (**b**,**e**) forming on pristine (no cell cycling and no exposure to electrolyte solution) LNO-based electrodes. **c**,**f**, Comparing simulations with bulk-sensitive (TFY-mode) Ni L-edge XAS experiments clarifies the true type of ligand-hole configuration in pristine LNO-based electrodes.

In Fig. 4, we begin with an electronic ground state assumed to have a nominal Ni^3+^ configuration (3*d*^7^) with explicit Ni–O hybridization. This represents a limiting single-site description in which hybridization is present but ligand-hole stabilization is not yet spontaneously developed. The mismatch between the simulated Ni L-edge spectrum and the experimental data confirms that such a purely local picture is insufficient to describe pristine LNO.

We, therefore, consider a ligand-hole description (that is, spontaneous charge transfer within a strongly hybridized Ni–O orbital). This description has a ${d}^{8}{\underline{L}}^{1}$ configuration as the dominant local motif in pristine LNO. In the literature, this picture is often introduced with implicit assumptions that are not always stated explicitly. First, the spatial extent of the hybridized orbital is assumed to be confined to a single NiO_6_ octahedron. Second, within this single-site framework, the ${d}^{8}{\underline{L}}^{1}$ state arises from hybridization between Ni e_g_ orbitals and the O ligands pointing directly towards the metal centre (Fig. 4a). These assumptions indicate a homogeneous lattice of equivalent NiO_6_ units, each hosting the same ligand-hole configuration (Fig. 4b). However, as shown in Fig. 4c, simulations based solely on this single-site ${d}^{8}{\underline{L}}^{1}$ picture reproduce the experimental Ni L_3_-edge spectrum poorly because these theoretical conditions do not consider the entire crystallographic structure.

A more complete description requires moving beyond single-site models towards collective electronic responses across the NiO_6_ network (Fig. 4d). When Ni3*d*–O2*p* hybridization extends over multiple octahedra, ligand holes can be stabilized through a self-regulating response mechanism^39^. In this picture, charge redistribution is not confined to a single site but is instead shared among neighbouring NiO_6_ units via ligand–metal bond reorganization, leading to configurations of the type illustrated in Fig. 4e. Such structural motifs have been observed experimentally in LNO^18,20^. This collective response results in coexisting configurations of the form ${d}^{8}{\underline{L}}^{0}$, ${d}^{8}{\underline{L}}^{1}$ and ${d}^{8}{\underline{L}}^{2}$, which correspond to distinct local NiO_6_ environments, with emerging evidence linking them to different local octahedral distortions. A statistical mixture of these configurations effectively reproduces the experimental Ni L-edge spectra (Fig. 4f), confirming that a self-regulating electronic structure provides a realistic description of pristine LNO. To capture the essential physics, we simulate the Ni L-edge response using three independent SIA models corresponding to these configurations. Although a fully quantitative description would require multi-site cluster calculations to simultaneously capture covalency, correlation and bond disproportionation^40,41^, the present approach provides a transparent physical interpretation of the dominant spectral features.

**Fig. 5:**
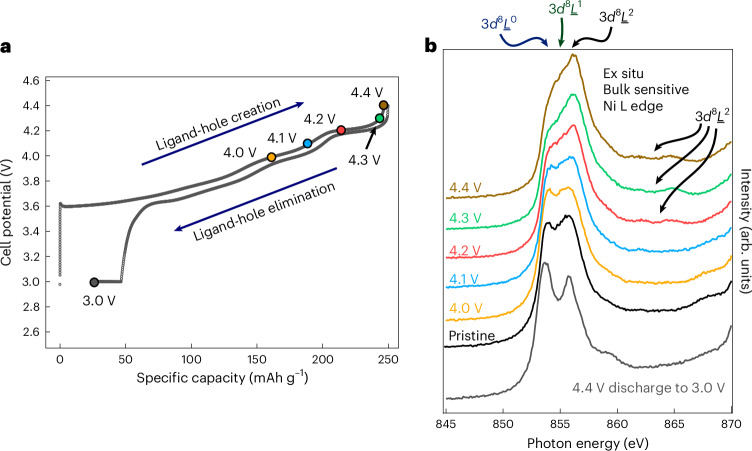
Ligand-hole evolution in LNO with charging and discharging. **a**, Electrochemical data of Li||LNO coin cells during *C*/20 (11 mA g^−1^) initial charge and discharge, with the circular shapes indicating cell potential points at which we stopped the measurements and disconnected the cells to carry out cell disassembly and electrodes harvesting for ex situ TFY mode Ni L-edge XAS studies. **b**, Ni L-edge in the TFY mode (bulk sensitive) of LNO-based electrodes harvested and sampled from cells disassembled at the given cell potentials (pristine means no cell cycling and no exposure to electrolyte).

Understanding the redox mechanism in LNO ultimately requires determining how the populations of ${d}^{8}{\underline{L}}^{0}$, ${d}^{8}{\underline{L}}^{1}$ and ${d}^{8}{\underline{L}}^{2}$ configurations evolve during lithiation and delithiation. To address this, we performed bulk-sensitive (TFY-mode) ex situ Ni L-edge XAS measurements on LNO electrodes cycled at different SoC in Li||LNO coin cells at *C*/20 (11 mA g^−1^) (Fig. 5a). From the analysis shown in Fig. 4f, the Ni L-edge spectrum can be decomposed into contributions from three main features at approximately 854, 855 and 856 eV, associated with the different local NiO_6_ environments and their corresponding ligand-hole configurations. On charging to 4.4 V, the intensity of the ${d}^{8}{\underline{L}}^{2}$ component increases, whereas the ${d}^{8}{\underline{L}}^{0}$ contribution decreases. This evolution indicates the progressive formation of ligand holes during charging, which are accommodated through redistribution among NiO_6_ units and increased Ni–O orbital hybridization, consistent with the trends observed in Fig. 2. This spectral evolution occurs within the Δ-dominated Ni L-edge regime (Fig. 5b) and is consistent with the DMFT-predicted increase in the ligand-hole population. Importantly, this establishes a direct link between electrochemical cycling and the redistribution of ligand-hole character within the hybridized Ni–O network. On discharge from 4.4 V to 3.0 V, the process reverses: ligand holes are progressively filled, reflected in the reduction of the ${d}^{8}{\underline{L}}^{2}$ component and the re-emergence of the ${d}^{8}{\underline{L}}^{0}$ contribution. This reversible evolution demonstrates that galvanostatic cycling modulates not only the oxidation state but also the statistical distribution of local NiO_6_ environments. As a consequence, electrochemical cycling induces a dynamic coexistence of distinct NiO_6_ motifs associated with ${d}^{8}{\underline{L}}^{0}$, ${d}^{8}{\underline{L}}^{1}$ and ${d}^{8}{\underline{L}}^{2}$ configurations, providing a direct spectroscopic signature of ligand-hole-mediated redox in LNO.

### Redox mechanisms and the Zaanen–Sawatzky–Allen scheme

**Fig. 6:**
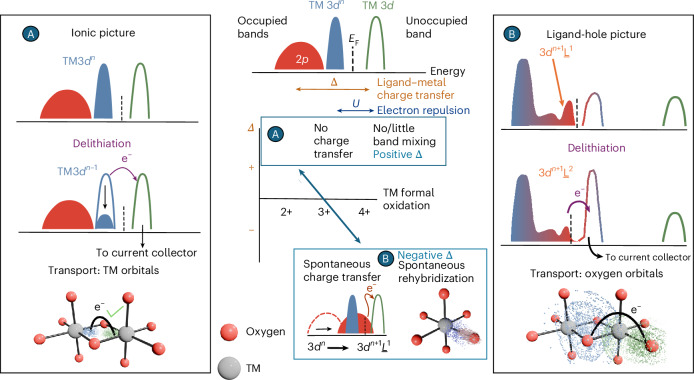
ZSA scheme and redox mechanisms. Schematics of the two end-point ZSA electronic models determined as a function of the relative strength of *U* and Δ. The ionic picture (model A) is the ZSA Mott–Hubbard material class with positive Δ and Δ > *U*. The ligand hole picture (model B) is the ZSA negative-charge-transfer material class with *U* > Δ, with positive *U* and Δ < 0. The type of gap between the electron conduction and valence bands in these models dictates the type of redox process during (de-)intercalation reactions. The way we understand the oxidation state evolution is then inherently linked to the relative strength of *U* and Δ.

The precise description of the electronic structures of LMFP64 and LNO have been critical for the distinct charge compensation pathways for these materials. We inferred their electronic structures using the Zaanen–Sawatzky–Allen (ZSA) electronic scheme. It was first discussed in ref. ^42^, where the relative energy positions of the metal *d* and oxygen *p* bands were used to categorize correlated TM compounds; a more recent work^43^ defines four categories based on the material’s relative energy values between Δ and *U*, which is the on-site Coulomb interaction, that is, the electron repulsion causing a separation in energy of the 3*d*-derived electronic states. Δ is defined similarly as in the RPES analysis. The material bandgap type formed within the ZSA electronic framework for LMFP64 and LNO dictates the corresponding charge compensation mechanism, illustrating how this bandgap can aid in determining the expected material redox nature in general (Fig. 6).

Model A in Fig. 6 describes the ZSA Mott–Hubbard material class—positive Δ (Δ > U)—applicable to the LMFP64 case. The bandgap results from Coulomb repulsion, so it is of a *d*–*d* nature. This means that the lowest unoccupied band (conduction band) and the highest occupied band (valence band) are both of the Fe*d* character. During cell charging, charge from the Fe3*d* valence band is promoted to the conduction band. The loss of Fe intensity in Fig. 1d is a manifestation of this effect and, hence, Fe oxidation. Model B in Fig. 6 defines the ZSA negative-charge-transfer material class, which determines the LNO electronic structure. Since in these materials, Δ is negative and *U* is positive, the bandgap is of a *dp*–*dp* type, as shown by our DMFT/RPES analysis (Figs. 2 and 3 and Supplementary Figs. 5 and 7). During the charging process of Li||LNO coin cells, electrons are promoted across this *dp*–*dp* bandgap, in a similar fashion as the spectral migration in Supplementary Fig. 5, creating holes in the hybridized ligand orbitals and drawing capacity. The LNO system instantaneously undergoes a correlated redistribution of charge, resulting in the reconfiguration of the ${d}^{8}{\underline{L}}^{0}$, ${d}^{8}{\underline{L}}^{1}$ and ${d}^{8}{\underline{L}}^{2}$ states during galvanostatic cycling of the cell. As a result, the electrochemical redox of LNO (and related Ni-rich positive electrodes) can be described by ligand-hole formation and annihilation (Fig. 5).

This ZSA-based redox framework has direct implications for the electronic structure governing redox mechanisms generally across the TM identity and formal oxidation state. Earlier TM compounds often exhibit weaker hybridization and less favourable ligand-hole stabilization at low-valent redox pairs, placing them on the Mott–Hubbard side of the ZSA classification, that is, conventional metal-centred redox. This is the case for the LMFP64 material studied here, and even for high-valence V, where in VOPO_4_, purely ionic V-centred redox drives the evolution to V^5+^ despite operation at 4.0 V (ref. ^44^). However, exotic oxidation states (≥3+) can progress through the ZSA stages and reach the negative-charge-transfer regime, where the ligand-hole behaviour emerges. This is exemplified in high-valence Fe (Fe^4+^ or higher), where reports indicate the onset of ligand-hole character^45^, and for which the ZSA framework can directly provide design rules to stabilize Fe ligand-hole states. By contrast, late TM compounds exhibit stronger hybridization, enabling ligand-hole characteristics at relatively lower oxidation states. For Co, once oxidation proceeds beyond Co^3+^, the system can evolve into the ZSA negative-Δ charge-transfer regime, where ligand-hole redox dominates (as observed for Co^3+^ → Co^4+^ in LiCoO_2_)^46^. Together with the Ni results presented here, this indicates that in mixed-metal cathodes such as NCA or NMC, the redox mechanism probably involves an interplay between Ni-driven ligand-hole formation and Co at the onset of its transition from mixed-valence to negative-Δ behaviour.

More broadly, this ZSA-based redox framework establishes a generalized redox strategy applicable to Li-excess compounds—the systems that originally motivated the study of oxygen redox. Note that the transition from positive- to negative-charge-transfer regimes depends on the metal and its formal oxidation state, corresponding to roughly Ni^2+^, Co^3+^ and Mn^4+^ (ref. ^43^).

## Conclusions

Here we present how by experimentally capturing the true nature of the electronic structure of LMFP64 and LNO positive electrode active materials under the ZSA scheme, the TM L-edge-assisted RPES approach can directly probe their corresponding redox mechanism. Using this framework, we can conclude that the degree of metal–oxygen hybridization is a key factor in determining the charge compensation mechanism. For more ionic compounds in the positive-charge regime, such as phosphates, electron counting of the *d* orbital remains valid (for example, Fe^2+/3+^ ≡ 3*d*^6^, 3*d*^5^). Increasingly covalent systems in the negative-charge-transfer regime, which includes LiMO_2_ (M = Co, Ni) family, are better described in terms of electron-counting ligand-hole states instead (for example, ${\rm{Ni}}^{3+/4+}\equiv {d}^{8}{\underline{L}}^{1}$, ${d}^{8}{\underline{L}}^{2}$). We expect this framework to also help describe Li-excess compounds. Most Li-excess compounds are Li rich or Mn rich^3^. Li_2_MnO_3_ and Li_2_NiO_3_ reflect the metal and ligand-hole end-points, respectively, consistent with the observed electrochemistry arising from structural and chemical decomposition^36,47^. Thus, we anticipate these materials could be classed in one of the other two ZSA categories (positive charge transfer or mixed valence) with varying *p*–*d* bandgap types^16^, offering a pathway to study the role of unhybridized oxygen orbitals in delivering reversible capacity without conflicting signals. We acknowledge that structural rearrangements also have a key role in the electrochemical behaviour of Li-rich systems^25^. TM L-edge-assisted RPES offers an opportunity to directly explore the electronic structures of Li-excess compounds under the ZSA scheme during (de)lithiation and to validate models and descriptions that clarify the role of oxygen oxidation in these compounds.

## Methods

### Ni-based materials preparation

#### LaNiO_2_

Single crystals of LaNiO_3_ in the perovskite phase were grown using the high-pressure optical floating zone method^48^. The crystals were oriented with X-ray Laue diffraction and cut into cube-shaped pieces with surface dimensions of approximately 1 mm^2^. Subsequently, oxygen was deintercalated via a direct-contact topotactic reduction with CaH_2_, transforming the perovskite phase into the LaNiO_2_ infinite-layer phase^49^. A thin decomposed layer on the surface of the reduced crystals was removed by mechanical polishing^50^. X-ray diffraction from the polished surface confirms a phase-pure, highly crystalline LaNiO_2_ composition.

#### NiO

Commercial powder material (99.99% trace metals basis) was purchased from Sigma-Aldrich (203882).

#### Li_2_NiO_3_

The Ni(OH)_2_ precursor was prepared through a precipitation reaction carried out in a stirred tank reactor (Eppendorf). A 2-M NiSO_4_ aqueous solution was pumped into an aqueous base solution of 0.4-M NH_4_OH within the reactor. Concurrently, separate solutions of 2-M NaOH (molar ratio of NaOH:Ni = 2) and NH_4_OH (NH_4_OH:TM = 1.2) were pumped into the reactor. A pH of 11 was maintained by the reactor by adjusting the NaOH flow rate. The reaction temperature was stirred for 20 h at 1,000 rpm, where the reactor temperature was maintained at 60 °C. The Ni(OH)_2_ precipitate was obtained after washing and drying at 80 °C overnight.

To obtain Li-rich Li_2_NiO_3_ powder, the solid-state preparation method reported previously^51^ was used. Stoichiometric amounts of Ni(OH)_2_ and LiOH⋅H_2_O were thoroughly mixed through hand grinding for 20 min in air using an agate mortar and pestle and transferred to a tube furnace. To form Li_2_NiO_3_, a preheating step at 300 °C for 12 h was first applied, followed by further heating at 550 °C for 24 h. All heating steps were performed under pure O_2_ (BOC 99.5%) flow and heating rates were set to 3 °C min^−1^. The resulting mixture was then ground with an agate mortar and pestle. Grinding took place for 5 min to break up loose aggregates, in the glovebox (H_2_O and O_2_ < 0.5 ppm) under Ar (BOC 99.998%) and stored in there before use. The material structure was previously confirmed to be phase-pure indexed by the *C*2/*m* space group^36^. After 2 days of storage in an inert-atmosphere glovebox, the samples were shipped to the testing facility at WMG. Following receipt, they underwent a 1-week conditioning period before measurement, resulting in a total elapsed time of 9 days between synthesis and characterization.

#### Single-crystal LNO

Single-crystalline LNO was prepared using Ni(OH)_2_, synthesized with the same method as for Li_2_NiO_3_.

A molten-salt-assisted method was used to obtain the single-crystal morphology. The Ni(OH)_2_ powder was finely ground in an agate mortar in air for 20 min with LiOH⋅H_2_O (Alfa Aesar 99.995%) and Li_2_SO_4_ (Sigma-Aldrich 98.5%) in a molar ratio of 1:1.5:0.25. The homogenized mixture was placed in an alumina crucible and subjected to a two-stage heat treatment in a tube furnace under O_2_ atmosphere. The first stage involved heating at 480 °C for 12 h, followed by the second stage at 775 °C for 24 h. After cooling, the product was washed with deionized water to remove residual Li species, recovered by centrifugation and subjected to a final heat treatment at 775 °C for 6 h under an O_2_ atmosphere to minimize surface degradation. The washing process was carried out as follows: the recovered crucibles were soaked in deionized water (18 MΩ) in an ultrasonic bath for 1 h to break up the brick-like product and recover the powder. The recovered powder was then added to a 50-ml centrifuge tube with 45 ml of deionized water. The centrifuge tube was then ultrasonicated for 30 s before centrifugation. The material was centrifuged at 8,000 rpm for 2 min, after which the supernatant was discarded. A further 45 ml of fresh deionized water was then added to the centrifuge tube and the process was repeated four times (for a total of five washes). After the final supernatant was discarded, the powder-containing centrifuge tubes were dried out in a vacuum oven at 80 °C for 16 h under a dynamic vacuum. All heating stages were performed at a ramp rate of 5 °C min^−1^. The material was removed from the furnace at 200 °C and immediately transferred to an Ar-filled (BOC 99.998%) glovebox (H_2_O < 0.5 ppm, O_2_ < 0.5 ppm at 20 °C) for storage to preserve its quality. The samples were immediately shipped to WMG. After 1 week, positive electrodes made of these materials conditioned with the potential of interest were sent for measurements at the Diamond Light Source (DLS) I09 beamline, following in-house material protection procedure for transportation and loading.

#### Commercial LNO

Commercial powder material (<3-μm particle size (Brunauer–Emmett–Teller) and ≥98% trace metals basis) was purchased from Sigma-Aldrich (757365).

All of these Ni-based materials were used during the SI36917 RPES beamtime at the I09 beamline of DLS.

### Single-crystal LNO electrode and cells

Only the synthesized single-crystal LNO material was used for electrode and cell manufacturing. The other Ni-based oxides were used in their as-synthesized form.

#### Slurry formulation and electrode preparation

The slurry formulation and casting of single-crystal LNO were conducted in a controlled dry-room environment (dew point, –45 °C). Three grams of active material were weighed and mixed with electron-conductive carbon additive (commercial-grade carbon black, C65, Imerys) and polyvinylidene fluoride binder (battery grade, Solef 5130) in a weight ratio of 90:5:5. The mixture was homogenized using a planetary centrifugal mixer (Thinky, ARE-250, O_2_ gas atmosphere) at 1,300 rpm for 5 min. Anhydrous *N*-methyl-2-pyrrolidone (NMP; 99% extra pure, Thermo Scientific Chemicals) (0.9 g) was then added to form a uniform slurry, followed by 15 min of mixing with the same conditions as before the NMP addition to achieve a solid content of 53%.

In the dry room, the slurry was coated onto a 15-μm-thick aluminium foil (Cambridge Energy Solutions) using a manually operated 260-μm doctor blade, ensuring uniform deposition. The coated electrodes were dried under a vacuum at 120 °C overnight, achieving a coat weight of 118.2 gsm. Calendering was performed using a two-roller compactor at 85 °C and a roller speed of 1 m min^−1^, resulting in a pressed density of 3.0 g cm^−3^ and an areal capacity of 2.57 mAh cm^−2^. The dry and calendered electrodes were cut using a 14.8-mm-diameter EL-cell electrode cutter. The final mass loading of the active material in the dry and calendered electrodes was 13.2 mg cm^−2^. The final average thickness of the dry and calendered electrodes was 50 μm.

#### Coin-cell assembly and electrochemical cycling

The assembly of Li metal CR2032-type coin cells was conducted in an Ar-filled glovebox (MBraun, O_2_ and H_2_O < 0.1 ppm). A Celgard 2325 Trilayer microporous membrane with a thickness of 25 μm and a diameter of 16 mm was used as a separator. Li discs (Cambridge Energy Solutions, battery grade, 99.2% purity, 0.3-mm thickness) for CR2032-type coin cells were used as the negative electrodes. Using an Eppendorf Research Plus pipette and pipette tips, the coin cells were filled with 60 μl of E151 Solvionic electrolyte solution (water content, <15 ppm; high purity, >99.9%), comprising 12.42:30.82:54.76:2 w/w ratio of LiPF_6_, ethylene carbonate, ethyl methyl carbonate and vinylene carbonate. The coin cells were crimped under 0.8 T with an electronically controlled MSK-160E crimper inside an Ar-filled (BOC 99.998%) glovebox (MBraun, O_2_ and H_2_O < 0.5 ppm). To ensure full wetting of the electrodes, the cells were held at rest at 25 °C for 20 h. Cells underwent one constant-current *C*/20 (*C* = 220 mA g^−1^) charge process to cell potentials (4.0, 4.1, 4.2, 4.3, 4.4 and 4.6 V) on a BioLogic VMP3 potentiostat cycler in a temperature-controlled chamber (25 °C). The corresponding active mass used to compute the specific capacities were 22.15, 23.01, 22.62, 22.5, 22.24 and 22.7 mg.

For each electrochemical condition presented (that is, each cut-off potential), we assembled and tested three independent coin cells. This approach was adopted to mitigate experimental uncertainties commonly associated with coin-cell assembly, particularly electrode misalignment, which can influence the overall cell performance. The electrochemical data shown in the main text represent the best-performing cell from each set of three. These representative cells were selected based on their electrochemical quality (for example, potential profile and capacity) and alignment with the expected redox behaviour of LNO at the corresponding SoC. Although variability was observed within some sets, the selected cells follow a consistent trend across the full electrochemical range, reinforcing that they each accurately represent their respective SoC (Supplementary Fig. 7a and Supplementary Note 5).

#### Cell disassembly, electrode harvesting and sampling

After electrochemical testing with a 1-h open-circuit potential time, the coin cells were transferred and carefully disassembled within an Ar-filled (BOC 99.998%) MBraun glovebox (O_2_ < 0.1 ppm, H_2_O < 0.1 ppm) to prevent exposure to atmospheric moisture. This guarantees no air exposure during cell handling after cycling. Once inside the Ar-filled glovebox, the coin cells were disassembled with an electronically controlled MSK-160E crimper inside the glovebox and positive electrodes were harvested with ceramic tweezers. The electrodes were subsequently washed thoroughly by adding one 20-μl drop of dimethyl carbonate (DMC; Sigma-Aldrich, anhydrous, 99% purity, water content of <20 ppm) on the electrode. Drying included letting the DMC to self-evaporate for 2 min. After washing and drying, the electrodes were placed in an airtight container provided by DLS and that was previously transferred into the glovebox and filled with the same Ar quality and purity. This airtight unit was transported to DLS and carefully loaded onto the sampling instrument, providing end-to-end protection of the electrodes against air exposure. This procedure was followed for the SI36917 and SI30201 RPES beamtimes and the SI33459 Ni L-edge beamtime.

### LiFePO_4_ and LMFP64 electrodes and cells

#### Slurry formulation and electrode preparation

LiFePO_4_ (LFP) and LMFP64 powders were purchased from Gelon LIB and used to fabricate the electrodes following in-house procedures. Graphite powder (BTR V-H) was purchased from Targray and Li from Cambridge Energy Solutions (battery-grade Li metal and discs). All powders were processed utilizing optimized protocols developed in-house based on supplier recommendations.

Electrodes were produced via slurry mixing (THINKY ARE-250) and casting (ERICHSEN COATMASTER 510). In a typical mixing process, LFP and LMFP64 active material and carbon black (Imerys C65) powders were weighed and mixed with polyvinylidene fluoride (Solef 5130) predissolved in 8-wt% anhydrous NMP (99.1% extra pure, Thermo Scientific Chemicals) to the desired ratio. LFP and LMFP64 utilized a formulation of 93:3.5:3.5 (AM/CB/polyvinylidene fluoride). LFP and LMFP64 were cast onto a carbon-coated Al foil (Cambridge Energy Solutions, thickness of 18 μm). For graphite electrodes, graphite powder, styrene–butadiene rubber (Zeon BM451) and carbon (Imerys C45) were mixed in air for 20 min with carboxymethyl cellulose (Ashland BVH8) predissolved in NMP (12 wt%) to the desired ratio in a weight ratio of 95.5:1.5:2.25:1 (graphite/carboxymethyl cellulose/styrene–butadiene rubber/carbon) ratio. This mixture was coated onto a 10.2-μm-thick copper foil (Avocet Steel Strip). The mixing for all these slurries was carried out in 5-min intervals at 1,300 rpm and gradually adding NMP (5 wt%) to adjust the solid content to approximately 59%. Final mixing of the slurry was performed for 15 min at 1,300 rpm, with a final defoaming step for 2 min at 1,300 rpm. Electrode coat weights were 135 gsm (13.5 mg cm^−2^) and 112 gsm (11.2 mg cm^−2^) for the LFP/LMFP64 and graphite electrode, respectively. These coat weights lead to NP ratios in the range of 1.1–1.2. All electrodes were dried overnight under a vacuum at 120 °C. Calendaring of the electrode sheet was carried out using a two-roller compactor at 85 °C, at a roller speed of 1 m min^−1^. Electrodes were calendered to the desired densities of 2.4 g cm^−3^ (LFP), 2.15 g cm^−3^ (LMFP64) and 1.5 g cm^−3^ (graphite). The final average thickness of the dry and calendered electrodes was 70 μm.

#### Cell assembly and electrochemical cycling

The assembly of Li CR2032-type coin cells using LMFP64 electrodes was conducted in an Ar-filled (BOC 99.998%) glovebox (MBraun, O_2_ and H_2_O < 0.1 ppm). A Celgard 2325 Trilayer microporous membrane of 25-μm thickness and 16-mm diameter was used as a separator. Li discs (Cambridge Energy Solutions, battery-grade, 99.2% purity, 0.3-mm thickness) for CR2032-type coin cells were used as the negative electrodes. LMFP64 positive electrodes (14.8-mm diameter) were cut using an EL-cell electrode cutter. Using an Eppendorf Research Plus pipette and pipette tips, the coin cells were filled with 100 μl of E151 Solvionic electrolyte solution (water content, <15 ppm; high purity, >99.9%), comprising 12.42:30.82:54.76:2 w/w ratio of LiPF_6_, ethylene carbonate, ethyl methyl carbonate and vinylene carbonate. The coin cells were crimped under 0.8 T with an electronically controlled MSK-160E crimper inside an Ar-filled (BOC 99.998%) glovebox (MBraun, O_2_ and H_2_O < 0.5 ppm). To ensure full wetting of the electrodes, the cells were held at rest at 25 °C for 20 h. The cells were then charged galvanostatically to a specified potential using a current of 7.9307 mA g^−1^ (equivalent to a *C*/20 charge rate with and average of 22 mg of active material). Specific capacity reported refers to the active material mass at 21.6, 21.8, 21.5, 22.1, 21.5 and 21.7 mg for pristine, 0%, 3.45%, 41.38%, 44.83% and 100% SoC, respectively (100% SoC of ~145 mAg h^−1^). LFP and LMFP64 pouch cells were assembled in a dry room (dew point, less than –40 °C) to prevent electrode moisture exposure, utilizing 1 g of electrolyte (1-M LiPF_6_ in 3:7 ethylene carbonate/ethyl methyl carbonate + 2% vinylene carbonate) and a Celgard 2325 separator. Graphite electrodes with the balancing (NP ratios) in the range of 1.1–1.2 were used as the counter electrodes, namely, the positive electrode dimensions were 48 mm × 68 mm and the negative electrode dimensions were 50 mm × 70 mm. The cells were allowed to soak for a period of 24 h, after which they underwent a formation process, consisting of two cycles at a rate of *C*/20 (*C* = 160 mA g^−1^). Following these formation cycles, cell underwent operando XAS Fe K-edge measurements. The operando cycling was carried out using a BioLogic SP150 cycler coupled with EC-lab software with *C*/3 cycles between 2.5 V and 4.5 V versus graphite. For calculations of cell capacity and *C* rate, a practical capacity of 160 mAh g^−1^ was assumed for both LFP and LMFP, that is, a rate of 1*C* indicates a current of 160 mA g^−1^.

#### Cell disassembly, electrode harvesting and sampling

After charging, the coin cells were carefully transferred and disassembled in an Ar-filled glovebox (O_2_ < 0.1 ppm, H_2_O < 0.1 ppm). The coin cells were disassembled with a electronically controlled MSK-160E crimper inside the glovebox and positive electrodes were harvested with ceramic tweezers, after which they were rinsed with DMC (Sigma-Aldrich, anhydrous, 99% purity, water content < 20 ppm) and dried. Drying included letting the DMC to self-evaporate for 2 min. After washing and drying, the electrodes were placed in an airtight container provided by DLS and that was previously transferred into the glovebox and filled with the same Ar quality and purity. This airtight unit was transported to DLS and carefully loaded onto the sampling instrument, providing end-to-end protection of the electrodes against air exposure. This material was used in the SI35075 RPES beamtime after careful sample loading and transportation that avoids air exposure.

### RPES

RPES data at Ni2*p*, O1*s* and Fe2*p* absorption thresholds were recorded at beamline I09 DLS across three sessions: Ni and O RPES in SI30201 and SI36917 beamtimes, and Fe RPES in SI35075 beamtime. For all these sessions, we used a VG Scienta EW4000 detector with a charge-coupled device camera at 70 fps. The four Ni-based materials in the form of a powder were used in this beamline. Li||LNO coin cells, as described earlier, charged to and opened at 4.2 V, 4.4 V, 4.6 V and discharged to and opened at 3.0 V were used in these beamlines. Additionally, LMFP electrodes (described earlier) and charged at 3.45%, 41.38%, 44.83% and 100% SoC were also used in this beamline. The total energy resolution for the measurements at I09 was <0.2 eV. The calibration of the photon energy was performed by comparing the kinetic energy of the Au4*f* peak. The base pressure during all the measurements was less than 2 × 10^−10^ torr and all the measurements were performed at room temperature.

### XAS L-edge TEY and TFY

Ni L-edge XAS measurements were performed in the TEY and TFY modes at the B07 beamline^52^ at DLS under the SI33459 beamtime. Energy calibration was performed using a NiO reference. The Ni spectra were collected with an energy resolution of <0.15. LNO material from Li||LNO coin cells, as described earlier, charged to and opened at 4.0, 4.1, 4.2, 4.3 and 4.4 V were used in this beamline.

### Operando XAS Fe K-edge

Operando Fe K-edge XANES measurements were performed at the X-Ray Diffraction Research Technology Platform (X-Ray Diffraction RTP), University of Warwick. We used an easyXAFS300+ spectrometer in the transmission mode with the instrument’s spherically bent crystal analyser for Fe K-edge energy range. X-ray air scattering was minimized using a helium gas chamber. The XANES data collection was optimized to last a total measurement time of approximately 14 min per scan with good data quality. Each dataset was dead-time corrected, normalized (using the empty beam) and energy calibrated using a reference Fe foil with the instrument software. The subsequent pre-edge background subtraction and post-edge normalization were carried out using the Athena software package. For each operando dataset, the pre-edge and normalization range values were optimized for the first scan and then fixed for the remaining scans. The half-height of the normalized spectra, that is, the energy value at which the intensity is 0.5, was calculated using the Athena software.

### XAS L-edge SIA simulations

Ni L-edge simulations were performed with a parameterized model of a single NiO_6_ octahedron (*O*_*h*_ point group), which contained Ni2*p*, Ni3*d* and ligand orbitals. The ligand orbitals were defined as linear combinations of O2*p* Wannier orbitals. The model Hamiltonian consisted of the Coulomb repulsion between (1) two Ni3*d* electrons (including all multiplet effects), (2) a Ni2*p* core and Ni3*d* valence electron (including all multiplet effects), (3) spin–orbit interaction in the Ni3*d* and Ni2*p* core level, (4) the on-site energy of the Ni2*p* core orbitals, (5) the orbital-dependent on-site energy of the Ni3*d* valence and ligand orbitals, and (6) the hybridization strength between the Ni3*d* and ligand orbitals. Using this Hamiltonian, XAS excitation was calculated using QUANTY (http://quanty.org/start), which calculates the spectra implementing Green’s function under the dipole approximation.

Parameter values enter our model in the form of Coulomb interactions, on-site energies, spin–orbit interactions and hopping integrals. The values for these parameters have been fairly well established over several decades of core-level spectroscopy and other techniques^29,42,53^. For the monopole Coulomb interaction parameters, we used Udd = 6 eV and Upd = 7 eV. For the ligand-field splitting, we used 10Dq = 0.95 eV between *d* orbitals and 10DqL = 1.44 eV between the Wannier ligand orbitals. For the intracluster hopping integrals, we used Veg = 3.0 eV and Vt2g = 1.74 eV. Spin–orbit interaction parameters were taken as the atomic values for Ni3*d*^7^, *ξ*2*p* = 11.3069 eV and *ξ*3*d* = 0.091 eV. Finally, the multipole Coulomb interaction parameters are taken as 80% of their atomic Hartree–Fock values for Ni3*d*^7^ (ref. ^54^): F2dd = 10.622, F4dd = 6.636, F2pd = 6.680, G1pd = 5.066 and G3pd = 2.882, all expressed in units of electronvolts. A charge-transfer energy (Δ) of 4.2 eV for ${d}^{8}{\underline{L}}^{0}$ states was selected and we used –1.2 eV and –2.6 eV for ${d}^{8}{\underline{L}}^{1}$ and ${d}^{8}{\underline{L}}^{2}$, respectively.

Fe L-edge simulations were also performed with a parameterized model of a single FeO_6_ octahedron (*O*_*h*_ point group), which contained Fe2*p* and Fe3*d*. No ligand orbitals are used as in the ionic picture. O ions only interact elctrostatically with the Fe3*d* orbitals resulting in the expected t_2g_ and e_g_ orbitals. The model Hamiltonian consisted of the Coulomb repulsion between (1) two Fe3*d* electrons (including all multiplet effects), (2) a Fe2*p* core and Fe3*d* valence electron (including all multiplet effects), (3) spin–orbit interaction in Fe3*d* and Fe2*p* core levels and (4) the orbital-dependent on-site energy of the Fe3*d* valence. Using this Hamiltonian, XAS excitation was calculated using QUANTY (http://quanty.org/start), which calculates spectra-implemented Green’s function under the dipole approximation.

Parameter values enter our model in the form of Coulomb interactions, on-site energies, spin–orbit interactions and hopping integrals. For the ligand-field splitting, we used 10Dq = 1.1 eV between the *d* orbitals. Spin–orbit interaction parameters were taken as the atomic values for Fe3*d*^6^, *ξ*2*p* = 8.2000 eV and *ξ*3*d* = 0.0520 eV. Finally, the multipole Coulomb interaction parameters are taken as 80% of their atomic Hartree–Fock values for Fe3*d*^6^ (ref. ^54^): F2dd = 9.8685, F4dd = 6.1335, F2pd = 6.1128, G1pd = 4.5000 and G3pd = 2.5587, all expressed in units of electronvolts.

### DMFT calculations

To obtain DFT-based Green’s functions as the starting point for our DFT + DMFT calculations, we used the full potential augmented plane-wave basis as implemented in WIEN2K^55^. For the WIEN2K calculations, we used the largest possible muffin-tin radii, and the basis-set plane-wave cut-off was defined by *R*_min_ ⋅ *K*_max_ = 9, where *R*_min_ is the muffin-tin radius of the O atoms.

DMFT calculations were performed using the TRIQS/DFTTools modules^56–58^ based on the TRIQS libraries^59^. We perform DMFT calculations in a basis set of projective Wannier functions as implemented in the dmftproj module of TRIQS. It was also used to calculate the initial occupancy of the correlated orbitals. A projection window of −10 eV to +26 eV was chosen. The large window of unoccupied bands was chosen to account for any hybridization between Ni*d* and O*p* orbitals in the higher-energy unoccupied bands, for more accurate charge projections within the *d*−*dp* model. All five Ni*d* orbitals have been treated in the impurity model, whereas the oxygen states have been taken into account as non-interacting.

The SIA model constructed by mapping the many-body lattice problem to a local problem of an impurity interacting with a bath was solved using the continuous-time quantum Monte Carlo algorithm in the hybridization expansion^60^, as implemented in the TRIQS/CTHYB module^61^. For each DMFT step, 150,000 × 128 cycles of warm-up steps and 1,500,000 × 128 cycles of measures were performed for the quantum Monte Carlo calculations. We performed one-shot self-consistent DFT + DMFT calculations, using a fully localized-limit-type double-counting correction^62^. We use a fully rotationally invariant Kanamori Hamiltonian parameterized by Hubbard *U* and Hund’s coupling *J*_H_, where we set the intraorbital interaction to $U{\prime} =U-2{J}_{{\rm{H}}}$. For our DMFT calculations, we used *U* values ranging from 6 to 9 eV and *J*_H_ = 0.5 to 0.75 eV to scan the full range of the metal–insulator transition. The insulating state was seen to appear at *U* = 7 eV and *J*_H_ = 0.5 eV and, hence, for $U{\prime} =6$ eV, which also matches with the previous values of $U{\prime}$ in the literature^13,26,63–65^. This value also leads to good agreement of DMFT with experimental results. Real-frequency self-energies have been obtained using the maximum-entropy method of analytic continuation, as implemented in the TRIQS/MAXENT module^66^. DMFT total and projected densities of states have been obtained from the real-frequency self-energies and the post-processing tools of DFTTools.

### Green’s-function-based simulation of O K-edge XAS

We used the FEFF10 code for the ab initio calculation of K-edge XANES. FEFF uses Green’s-function-based formulation of the multiple scattering theory to compute the spectra^67,68^. The X-ray absorption *μ* is calculated in a manner similar to Fermi’s golden rule when written in terms of the projected photoelectron density of the final states or the imaginary part of the one-particle Green’s function, $G(r,{r}^{{\prime} };E)$. In terms of Green’s function $G(r,{r}^{{\prime} };E)$, the absorption coefficient *μ* from a given core level *c* is given by ref. ^69^.

$$\mu =-\frac{1}{{\rm{\pi }}}{\rm{I}}{\rm{m}}\langle c| {\epsilon }_{r}G(r,{r}^{{\prime} };E){\epsilon }_{{r}^{{\prime} }}| c\rangle ,$$

with Green’s function given by

$$G(r,{r}^{{\prime} };E)=\mathop{\sum }\limits_{{\rm{f}}}\frac{{{\Psi }}_{{\rm{f}}}(r){{\Psi }}_{{\rm{f}}}^{* }({r}^{{\prime} })}{E-{E}_{{\rm{f}}}+{\rm{i}}{\Gamma }},$$

where Ψ_f_ are the final states, with associated energies *E*_f_ and net lifetime Γ, of a one-particle Hamiltonian that includes an optical potential with appropriate core-hole screening. The FEFF code computes the full propagator *G* incrementally using matrix factorization and uses the spherical muffin-tin approximation for the scattering potential. For self-consistent potential calculations required in the XANES calculation for the Fermi level *E*_0_ estimation, a large value of rfms1 (radius of the cluster considered during the full multiple scattering calculation within the self-consistent field loop) was chosen to be 9 Å, to have a large number of atoms included in the self-consistent potential calculations. Full multiple scattering is required in the XANES calculation, as the multiple scattering expansion’s convergence might not be stable in the XANES calculation. A large rfms (radius of sphere centred on the absorbing atom (real space) or for the unit cell of the crystal (*k* space) to compute full multiple scattering calculations) value was considered to be 11 Å for proper convergence. The Hedin–Lundqvist self-energy was chosen for the exchange–correlation potential model used for the XANES calculation. The random phase approximation is used to approximate the core-hole interactions in our K-edge XANES calculations. The default experimental broadening of 0.3 eV given by FEFF was applied.

It is to be noted that the spectral lineshapes obtained from FEFF are found to be consistent with core-hole DFT spectral calculations using VASP6 (ref. ^70^); however, FEFF is more accurate with the calculation of edge energies.

## Online content

Any methods, additional references, Nature Portfolio reporting summaries, source data, extended data, supplementary information, acknowledgements, peer review information; details of author contributions and competing interests; and statements of data and code availability are available at 10.1038/s41565-026-02189-y.

## Supplementary information

Supplementary Information Supplementary Figs. 1–7, Supplementary Notes 1–5 and Supplementary References.

## Data Availability

All dataare available via Figshare at 10.6084/m9.figshare.28915994 (ref. ^71^).
