# Supplementary material for: Direct evidence of metal–ligand redox processes in positive electrodes during lithium-based battery operation
Source: Nat Nanotechnol. 2026 Jun 9;21(7):957–66. doi: 10.1038/s41565-026-02189-y (PMC13379316; doi:10.1038/s41565-026-02189-y)
Supplement: Supplementary file 1 — Supplementary Figs. 1–7, Supplementary Notes 1–5 and Supplementary References. [file 41565_2026_2189_MOESM1_ESM.pdf]

nature nanotechnology

Supplementary information https://doi.org/10.1038/s41565-026-02189-y

Direct evidence of metal–ligand redox processes in positive electrodes during lithium-based battery operation

In the format provided by the authors and unedited

Supplementary Information

Supplementary Figure 1-7

Supplementary Notes 1-5

References

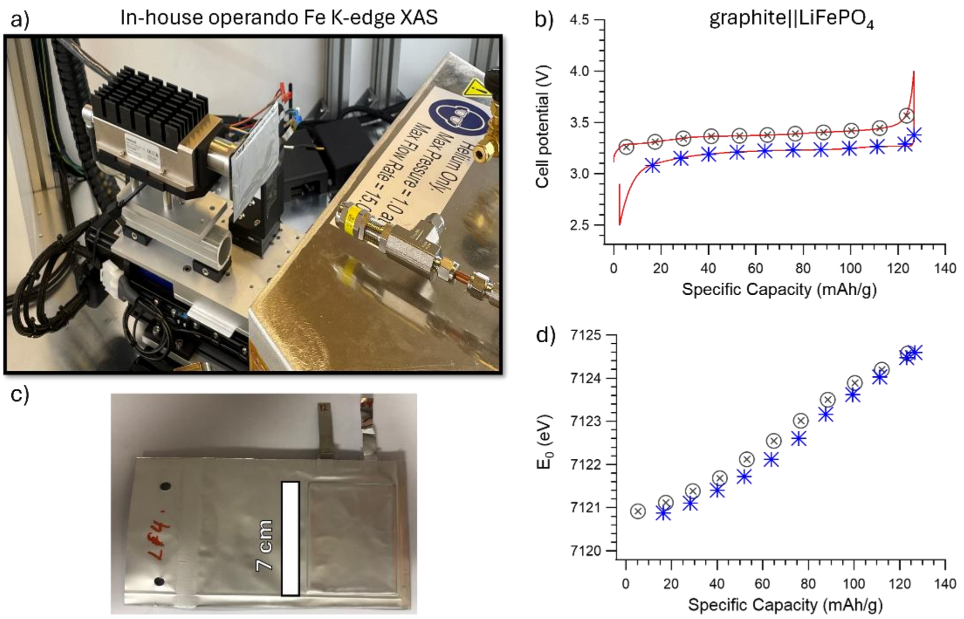

Supplementary Fig. 1: **Discussed in Supplementary Note 1.** a) In-house hard XAS setup. Retrieved with permission from American Physical Society under the terms of the Creative Commons Attribution 4.0 International license. Adapted from Ref. 1. With this, we measured Fe K-edge directly on a graphite||LiFePO4 pouch cell. b) Electrochemistry data of the graphite||$LiFePO_4$ pouch cell during the operando XAS studies. Marks represent the average cell potential and specific capacity of the corresponding Fe XAS scan reported in panel d. c) Photographic picture of a graphite||$LiFePO_4$ pouch cell produced in the WMG pilot line. d) Energy shift of the Fe absorption edge as a function of cell capacity for the graphite||$LiFePO_4$ pouch cell.

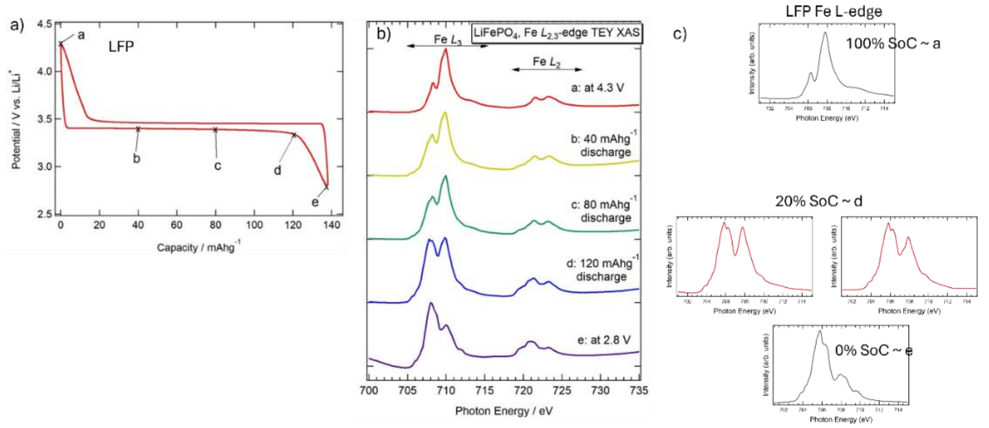

Supplementary Fig. 2: **Discussed in Supplementary Note 1.** a) Electrochemistry data of the Li||$LiFePO_4$ coin cell and b) corresponding Fe L-edge measurements in the TEY mode at the labelled points in panel a. Retrieved with permission from John Wiley and Sons 2026. Adapted from Ref. 4. c) Fe L-edge measurements in the TEY mode of the $LiFePO_4$ (LFP) electrodes we tested in graphite||$LiFePO_4$ coin cells at the specified state of charge (SoC). A qualitative comparison of the graphs in panel c and those in panel b shows similarities in the results.

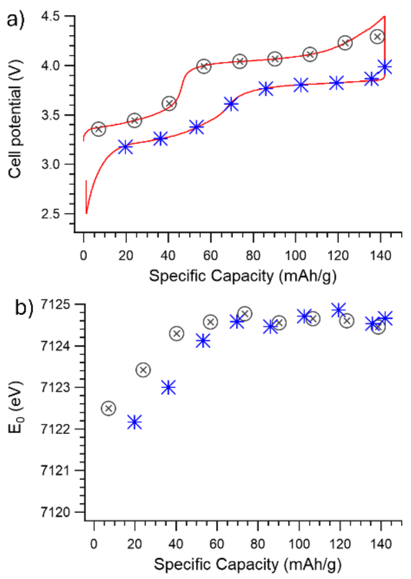

Supplementary Fig. 3: **Discussed in Supplementary Note 1.** a) Potential profiles of the graphite||$LiMn_{0.6}Fe_{0.4}PO_4$ pouch cell during the operando XAS studies. Marks represent the average cell potential and capacity of the corresponding Fe XAS scans reported in panel b. b) Energy shift of the Fe absorption edge as a function of cell capacity for the graphite||$LiMn_{0.6}Fe_{0.4}PO_4$ pouch cell.

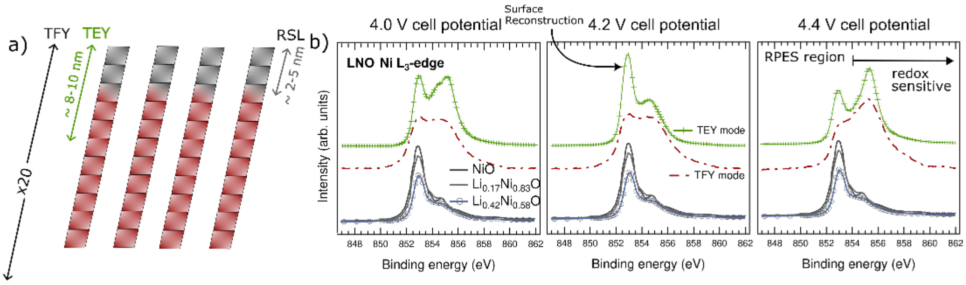

Supplementary Fig. 4: **Discussed in Supplementary Note 2.** a) schematic illustrating the comparison of the probing depths between the total fluorescence yield (TFY) mode and total electron yield (TEY) mode. It also shows the average expected thickness of the region that undergoes surface reconstruction as a result of electrochemistry. b) TEY and TFY of Ni L-edge on $LiNiO_2$ material harvested from Li||$LiNiO_2$ coin cells stopped at 4.0, 4.2, and 4.4 V. Ni L-edge reference in the TEY mode are also included for comparison of spectral fingerprints of expected surface phases: NiO, $Li_{0.17}Ni_{0.83}O$, $Li_{0.42}Ni_{0.58}O$. The Ni L-edge spectra of NiO, $Li_{0.17}Ni_{0.83}O$, $Li_{0.42}Ni_{0.58}O$ are reproduced from Ref. 2 with permission from the Royal Society of Chemistry.

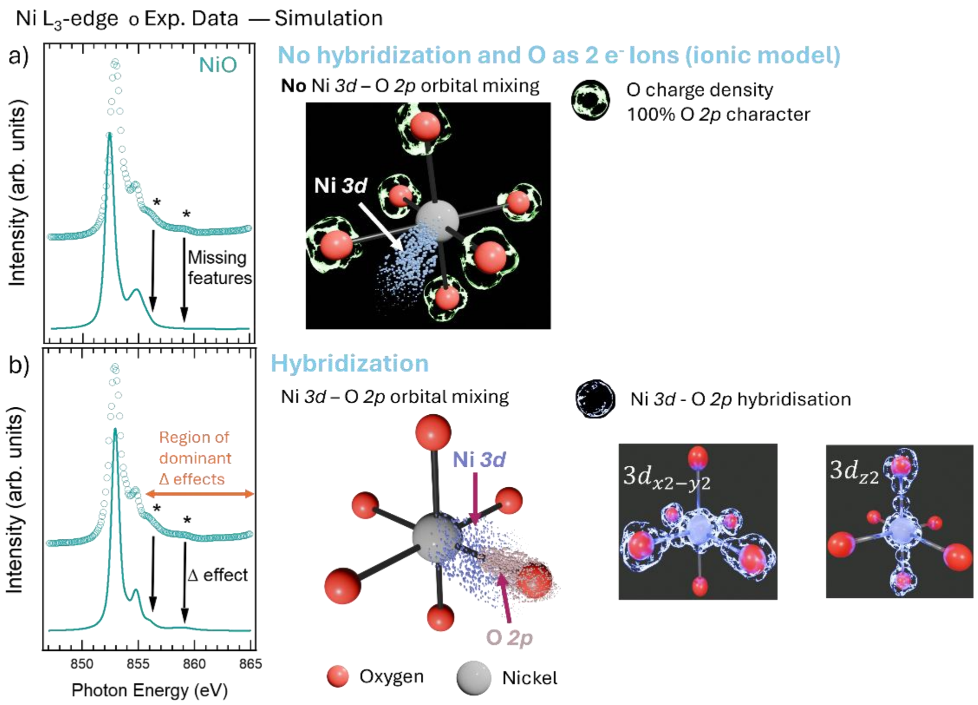

Supplementary Fig. 5: **Discussed in Supplementary Note 3.** Comparison of the effects of explicitly including Ni 3d – O ligand 2p orbital hybridisation in the Ni L-edge simulation. The case of no direct Ni 3d – O 2p hybridisation in a) represents the ionic model which underrepresents spectral features at 856 and 859 eV. The case in b) accounts for the explicit hybridisation of these orbitals allowing for some charge transfer to occur which reproduces the missing spectral features.

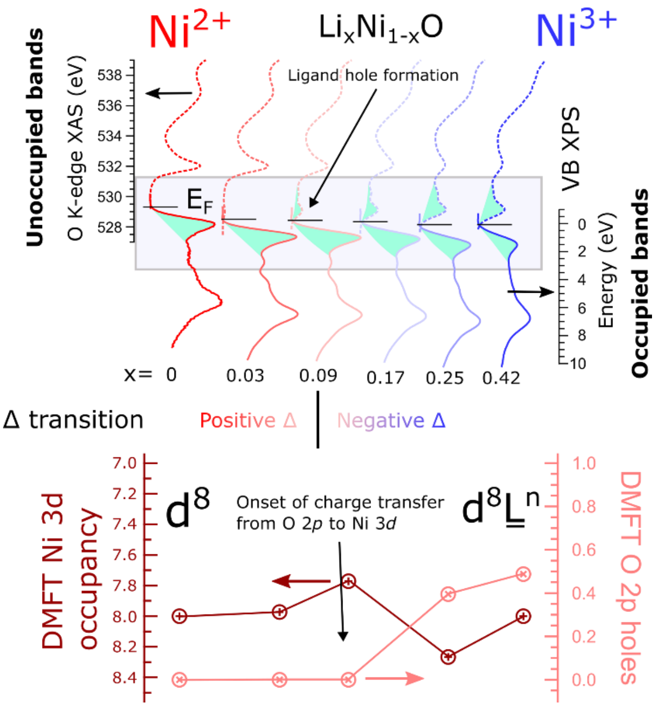

Supplementary Fig. 6: **Discussed in Supplementary Note 4.** Experimental and computational evidence of the transition of a system from positive to negative Δ resulting in the formation of stable ligand holes in the negative-Δ regime and a transition threshold in between Ni^{2+} and Ni^{3+} systems. Data from O K-edge (TEY mode) and XPS in the valence band (VB) region is reproduced from Ref. 2 with permission from the Royal Society of Chemistry.

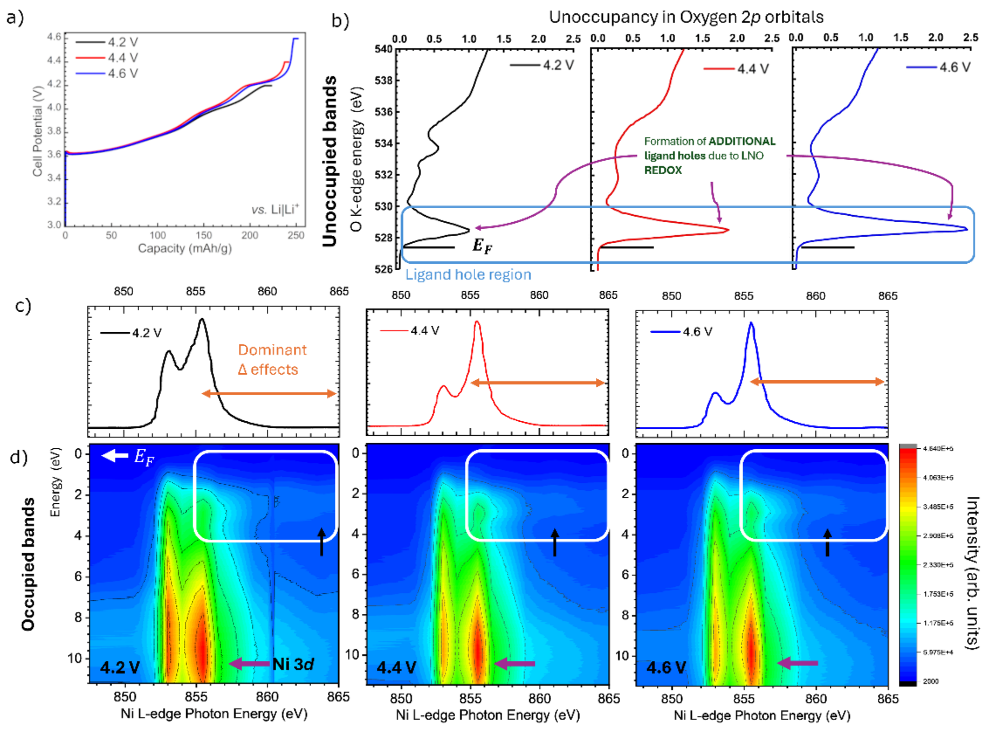

Supplementary Fig. 7: **Discussed in Supplementary Note 5.** a) Electrochemical data of the Li||LNO cell during C/20 (C = 220 mA/g) charge and stopped at different upper cut-off cell potentials for cell disassembly for RPES studies. b) Unoccupied O 2p orbitals in O K-edge experiments with a peak whose intensity increases due to ligand hole formation in the LNO positive electrode active material during cell charging at various upper cut-off potentials. c) Ni L-edge XAS measurements in the TEY mode of the LNO positive electrodes harvested after cycling in Li metal coin cells applying various upper cut-off potentials (4.2, 4.4, and 4.6 V) to identify X-ray photon energies for the RPES studies. d) Heat map showing the intensities of the RPES experiments indicating the energies of occupied orbitals and a black arrow showing the effects of additional ligand hole creation as a result of the charging process of the LNO material.

Supplementary Note 1: Interpretation of Fe L-edge spectral evolution in TEY mode

The Fe L-edge X-ray absorption spectra discussed in our research work probe transitions into Fe 3d-derived states and are therefore sensitive to the distribution of Fe d^n configurations and their associated multiple final states in Fe L-edge transitions. As a consequence, the magnitude and line-shape evolution of Fe L-edge spectra do not necessarily scale linearly with extracted capacity or with the bulk-averaged Fe oxidation state inferred from hard X-ray absorption measurements.

This distinction is illustrated in Supplementary Figs. 1 and 2 for cells containing $LiFePO_4$ (LFP). Operando Fe K-edge XAS collected on graphite||LFP pouch cells (Supplementary Fig. 1) shows a largely linear evolution of the Fe oxidation state across the electrochemical plateau, consistent with progressive changes in Fe–O bond lengths and bulk oxidation. In contrast, Fe L-edge spectra (Supplementary Fig. 2)—acquired on the same material harvested from graphite||LFP CR2032-type coin cells with the same electrolyte solutions as in the pouch cell counterpart—show comparatively limited spectral evolution both in prior literature[3,4] and in the present work over most of the plateau, followed by a more pronounced transformation near one end of the Fe oxidation region. Namely, Fe L-edge spectra in LFP remain largely unchanged across much of the plateau and evolve more strongly near the electrochemical endpoint.

For LMFP, a distinct behaviour is observed. Operando Fe K-edge XAS (Supplementary Fig. 3) reveals abrupt changes in the rising-edge region within the Fe redox window that coincide with the trends identified by the Fe L-edge spectra. This correspondence indicates that the emergence of the ~706 eV feature in the Fe L-edge spectra (Figure 1 main text) reflects an intrinsic, non-linear response of the Fe electronic structure during oxidation in LMFP, rather than an artefact arising from sample inhomogeneity or experimental limitations.

Taken together, the combined hard and soft X-ray absorption data demonstrate that while Fe oxidation in LFP proceeds smoothly at the bulk level, Fe L-edge spectral evolution can be concentrated near electrochemical endpoints due to the sensitivity of soft XAS to changes in Fe 3d electronic configurations. In LMFP, both operando hard XAS and ex situ soft XAS consistently reveal a pronounced, system-specific transition within the Fe redox region that is absent in LFP, supporting the interpretation of an intrinsic electronic reconfiguration associated with Fe oxidation in this material.

Supplementary Note 2: RPES energy region in Ni L-edge

Resonant photoemission spectroscopy (RPES) provides insight into the character of valence orbitals involved in redox processes through enhancement of spectral intensity at absorption resonances. In our RPES experiments guided by Ni L-edge XAS in total electron yield (TEY) mode, the choice of incident photon energy is critical to spectroscopically enhance signal for the orbital of interest.

When the photon energy coincides with an absorption feature dominated by surface species—such as the rock-salt-like (RSL) reconstruction—the resulting RPES signal primarily reflects surface characteristics. In contrast, selecting photon energies corresponding to redox-active bulk states preferentially enhances bulk-like orbital contributions. To determine suitable photon energies for probing bulk redox behaviour in our RPES studies, we first analysed Ni L-edge X-ray absorption spectroscopy (XAS) spectra collected in both TEY and total fluorescence yield (TFY) modes.

Using the NIST Electron Inelastic-Mean-Free-Path Database (version 1.2), we estimate a probing depth of ~8–10 nm for TEY-mode RPES at the Ni L_3 edge. Transmission electron microscopy (TEM) studies have shown that the RSL layer in cycled $LiNiO_2$ (LNO) typically ranges from ~2 to 5 nm in thickness.[5] Therefore, the TEY signal captures contributions from both the surface RSL and the adjacent subsurface region, as illustrated in Supplementary Fig. 4a. To disentangle surface and bulk contributions, we used TFY-mode XAS, which has an approximately 20× greater probe depth than TEY, to identify photon energies more representative of bulk redox activity originating from the subsurface region.

As shown in Supplementary Fig. 4b, the peak at ~853 eV is prominent in TEY but strongly attenuated in TFY, suggesting a surface-dominated origin. In contrast, the spectral region above 854 eV exhibits stronger intensity in TFY and tracks redox changes between 4.0 V, 4.2 V, and 4.4 V—indicating that it originates from bulk redox-active states. This interpretation is further supported by reference spectra: NiO (a proxy for the RSL phase) and $Li_xNi_{1-x}O$ (associated with transition-metal/lithium intermixing) both show dominant features near 853 eV.

These comparisons reinforce the assignment of the 853 eV region to surface species. Accordingly, our RPES experiments were conducted using photon energies >854 eV to enhance valence-band features associated with redox-active orbitals from subsurface regions, which are more representative of the bulk redox mechanism.

Supplementary Note 3: The Ni L-edge simulations and the spectra signatures of charge transfer

Supplementary Fig. 5 presents our simulations of the Ni L-edge XAS spectra for NiO using a single-octahedron model without (Supplementary Fig. 5a) and with (Supplementary Fig. 5b) explicit Ni *3d*–O ligand *2p* hybridisation. The hybridisation is implemented within a second quantization formalism, where Ni *3d* orbitals are coupled to symmetry-adapted linear combinations of O *2p* Wannier-like functions that match the symmetry of the Ni *3d* states.[6]

A key advantage of using these Wannier-like ligand orbitals—rather than a simplified ligand-field description—is their ability to better represent the spatial distribution of O *2p* charge density in a solid-state environment within a local octahedral model. This spatial fidelity enhances the realism of the hybridisation scheme.

When hybridisation is included (Supplementary Fig. 5b), the spectral features between 856 and 859 eV are well reproduced. In this case, although the charge-transfer energy Δ was set positive to suppress spontaneous ligand-to-metal charge transfer, a small charge transfer (~0.3 e^-) still occurs. This is not a spontaneous process but arises from induced X-ray transitions involving neighbouring O ions. These excitations correspond to a well-known charge-transfer mechanism forming a Zhang-Rice–like bound state.[7]

NiO is a system with positive Δ, meaning charge transfer is not energetically favoured and requires external excitation. However, in materials like $LiNiO_2$ and Li_2NiO_3, where the Ni oxidation state exceeds 2+, Δ becomes negative. These are therefore archetypal negative-Δ systems, in which ligand-to-metal charge transfer occurs spontaneously. Consequently, a more pronounced spectral contribution is expected in the Ni L-edge energy range above 855 eV. As a result, RPES measurements resonating in this energy region intensify spectral signals that reflect hybridisation-driven charge-transfer effects in nickel oxides with oxidation states above 2+.

Supplementary Note 4: The threshold from positive to negative Δ

A series of pulse laser deposition (PLD)-grown thin films with varying Li content simulate the progressive oxidation from Ni^{2+} in NiO to Ni^{3+} in $Li_xNi_{1-x}O$ as x increases from 0 to 0.5.[8] Using these materials, we computed using DMFT the orbital occupancies across the series and identified that the onset of charge transfer between Ni *3d* and O *2p* orbitals occurs at x = 0.09 (Supplementary Fig. 6). Beyond this point, the O *2p* hole occupancy increases continuously, indicating that the oxidation from Ni^{2+} to Ni^{3+} is primarily driven by ligand hole formation rather than depopulation of the Ni 3d orbitals.

This evolution can be observed experimentally by comparing the occupied and unoccupied states near the Fermi level (E_F). Valence band (VB) XPS reveals the occupied states, while O K-edge XAS (collected in total electron yield mode) probes the unoccupied O *2p* orbitals. As with the DMFT results, we observe at x = 0.09 the onset of a spectral shift from the occupied VB states to unoccupied O *2p* states just above E_F, followed by a continuous growth in unoccupied O *2p* intensity with further Li incorporation (Supplementary Fig. 6). This spectral shift provides direct experimental evidence of charge being removed from the O *2p* band, resulting in the formation of stable ligand holes $\underline{L}$.

The increasing O *2p* spectral weight above E_F confirms that Ni^{3+} formation is fundamentally associated with ligand-hole character—consistent with prior findings (Refs. [9] and [10]). This observation, together with our DMFT simulations, supports the conclusion that Ni *3d* and O *2p* orbitals are hybridised even in NiO. As the oxidation state increases beyond Ni^{2+}, the system transitions into the negative charge-transfer regime, characterised by strong Ni *3d*–O ligand *2p* hybridisation and spontaneous ligand-hole formation.

Supplementary Note 5: Li||LiNiO2 coin cell performance consistency

The variability described in the Methods section refers to the lack of complete overlap in the cell potential–capacity curves measured across upper cut-off potentials and different cells. This behaviour is illustrated in Supplementary Figure 7a, where a small but discernible vertical displacement between the curves reflects inherent cell-to-cell statistical variability.

To verify that these differences do not compromise the assignment of electrochemical states, O K-edge characterisation of $LiNiO_2$-based positive electrodes harvested at the corresponding potentials was performed. The spectra (Supplementary Figure 7b) show a systematic increase in pre-edge intensity with increasing state of charge, consistent with $LiNiO_2$ redox activity and the formation of ligand holes.

These observations confirm that, despite minor statistical variations between cells, the electrochemical states are reliably defined and follow a consistent trend across the full electrochemical window.

References

1. Menon, A. S. *et al.* Quantifying Electrochemical Degradation in Single-Crystalline LiNi0.8Mn0.1Co0.1O2–Graphite Pouch Cells through Operando X-Ray and Postmortem Investigations. *PRX Energy* **3**, 013004 (2024).
2. Zhang, J. Y. *et al.* Electronic and transport properties of Li-doped NiO epitaxial thin films. *J. Mater. Chem. C* **6**, 2275–2282 (2018).
3. Liu, X. *et al.* Distinct charge dynamics in battery electrodes revealed by in situ and operando soft X-ray spectroscopy. *Nat. Commun.* **4**, 2568 (2013).
4. Asakura, D. *et al.* Large Charge-Transfer Energy in LiFePO 4 Revealed by Full-Multiplet Calculation for the Fe L 3 -edge Soft X-ray Emission Spectra. *ChemPhysChem* **19**, 988–992 (2018).
5. Kim, M. *et al.* Improving LiNiO 2 cathode performance through particle design and optimization. *J. Mater. Chem. A* **10**, 12890–12899 (2022).
6. Haverkort, M. W., Zwierzycki, M. & Andersen, O. K. Multiplet ligand-field theory using Wannier orbitals. *Phys. Rev. B* **85**, 165113 (2012).
7. Lechermann, F., Körner, W., Urban, D. F. & Elsässer, C. Interplay of charge-transfer and Mott-Hubbard physics approached by an efficient combination of self-interaction correction and dynamical mean-field theory. *Phys. Rev. B* **100**, 115125 (2019).
8. Zhang, J. Y. *et al.* Electronic and transport properties of Li-doped NiO epitaxial thin films. *J. Mater. Chem. C* **6**, 2275–2282 (2018).
9. Green, R. J. & Sawatzky, G. A. Negative Charge Transfer Energy in Correlated Compounds. *J. Phys. Soc. Japan* **93**, 1–11 (2024).
10. van Elp, J., Eskes, H., Kuiper, P. & Sawatzky, G. A. Electronic structure of Li-doped NiO. *Phys. Rev. B* **45**, 1612–1622 (1992).
